# Lactate Overload Inhibits Myogenic Activity in C2C12 Myotubes

**DOI:** 10.1515/biol-2019-0004

**Published:** 2019-03-20

**Authors:** Sarah Se-Jung Oh, Sujin Kim, Sohee Moon, Dong-Ho Park, Ju-Hee Kang

**Affiliations:** 1 Department of Pharmacology and Medicinal Toxicology Research Center, Hypoxia-related Diseases Research Center, Inha University School of Medicine, Room 1015, 60th Anniversary Hall, 100, Inha-ro, Nam-gu, Incheon 22212, Republic of Korea; 2 Department of Kinesiology, Inha University, 100, Inha-Ro, Nam-gu, Incheon 22212, Republic of Korea; 3 Korea International School, Pangyo, Republic of Korea; 4 Department of Kinesiology, Inha University Incheon, South Korea; 5 Department of Pharmacology, Hypoxia-related Disease Research Center, College of Medicine, Inha University, Incheon, Republic of Korea

**Keywords:** Lactate, Skeletal muscle, Myogenesis, Akt, AMPK

## Abstract

Lactate (LA), an endogenous metabolite produced from pyruvate, can accumulate in skeletal muscle in certain conditions including major diseases, as well as during intensive exercise. Using differentiated C2C12 myotubes, we evaluated the early (1-h) and delayed (24-h) effects of LA (8 mM) on mechanisms involved in myogenesis or muscle atrophy, including 5'-adenosine monophosphate-activated protein kinase (AMPK)-mediated inhibition of protein synthesis through the mTOR/P70-S6K pathway, Akt-mediated inhibition of expression of the MAFbx atrophic factor by FOXO3a and expression of the myogenic transcription factors, MyoD, myogenin and myosin heavy chain. Although the early effects of LA overload were not significant on myogenic or atrophic mechanisms, LA treatment for 24 h significantly activated atrophic mechanisms but suppressed myogenesis in myotubes. In addition, LA overload for 24 h significantly suppressed the expression of Sirtuin 1 and peroxisome proliferator-activated receptor gamma coactivator-1 alpha. Consistent with LA-induced activation of atrophic mechanisms, the diameter of C2C12 myotubes treated with LA for 24 h, but not for 1 h, was significantly lower than in control myotubes. Thus, a sustained, but not a transient, LA overload could induce muscle atrophy through the regulation of AMPK- and Akt-mediated pathways, although further in vivo studies are needed to confirm this.

## 1 Introduction

Skeletal muscle is a dynamic organ system that plays important roles in maintaining whole body metabolism by the uptake and utilization of glucose and fatty acids. Adequate oxygen supply is essential when skeletal muscle uses glucose as an energy source—particularly during exercise — to generate ATP through mitochondrial oxidative phosphorylation. When oxygen requirement exceeds supply, the intracellular NADH/NAD^+^ ratio increases followed by the conversion of pyruvate to lactate (LA). Through the Cori (or lactic acid) cycle, LA is converted to glucose in the liver and is used to supply glucose to other organs, including skeletal muscle. However, when LA conversion is diminished, LA can accumulate in skeletal muscle. During acute- and high-intensity exercise, the rate of production of LA exceeds the conversion rate, so LA concentrations in skeletal muscle are over 10 mM [1, 2]. However, the concentration of LA in skeletal muscle and blood decreases rapidly after exercise with a half-life of <1 h [3]. Therefore, the concentration of LA in skeletal muscle in healthy individuals is rapidly decreased to its baseline concentration of <1 mM in the resting state within a few hours. However, in certain diseases, such as cancers or hepatic disease, LA can be present at high concentrations for longer times [4, 5] and this can induce lactic acidosis; drastic skeletal muscle atrophy is observed in these patients. The skeletal muscle atrophy seen in patients with chronic major diseases might be induced by coexistent inflammation [6, 7, 8]; however, the effects of LA overload on myogenesis has not been elucidated.

The production of LA is closely related to the type, intensity and duration of exercise [2]. During strenuous exercise in healthy persons, 75% of the LA produced is used as an energy source in skeletal muscle and 25% is provided for gluconeogenesis in the liver for continuous glucose supply via the LA shuttle [9, 10]. Skeletal muscle is a dynamic organ system in which various signaling proteins are involved in myogenesis and muscle atrophy. For example, protein kinase B (Akt) is a kinase phosphorylating the protein forkhead box class O 3a (FOXO3a), which is a transcription factor for the muscle atrophic signal, MAFbx [11]. When the activity of Akt is inhibited, dephosphorylated cytosolic FOXO3a is translocated into the nucleus and induces the expression of MAFbx. 5′-Adenosine monophosphate-activated protein kinase (AMPK) is another enzyme regulating myogenesis as well as glucose and fatty acid metabolism. AMPK can inhibit the maintenance of muscle mass through the activation of autophagy signals, but also acts in the inhibition of protein synthesis via the mammalian target of rapamycin (mTOR) signaling pathways [12, 13]. Activation of AMPK phosphorylates regulatory associated protein of mTOR (raptor), a scaffold protein of mTOR complex 1, which subsequently inhibits P70-S6 kinase (P70-S6K) and protein synthesis [14]. It has been reported that AMPK contributes to FOXO-mediated MAFbx expression followed by muscle atrophy, although contradictory results—that AMPK activation mediates the expression of the myogenic markers MyoD and myogenin [15] — have been reported [16]. Mitochondrial function is another potential contributor of myogenesis [17], and it is well known that peroxisome proliferator-activated receptor gamma coactivator 1α (PGC-1α) is a master regulator of mitochondrial biogenesis [18] through the activation by sirtuin1 (Sirt1)-mediated deacetylation [19]. Skeletal muscles are not static organs but respond to external and internal stimuli, and in which the levels of LA are also regulated dynamically. However, the effects of LA on several myogenic regulators are not fully elucidated. In this study, we test if the high concentration of LA that is observed during high-intensity exercise, or in pathological conditions of LA accumulation, regulates myogenic signaling pathways using differentiated C2C12 murine myotubes.

## 2 Materials and Methods

### 2.1 Cell culture and treatment

C2C12 mouse myoblasts were obtained from the American Type Culture Collection (ATCC; Manassas, VA, USA) and grown in high glucose Dulbecco’s Modified Eagle’s Medium (DMEM, Thermo Fisher Scientific, Waltham, MA, USA) containing antibiotics (100 U/mL penicillin and 100 μg/mL streptomycin), 10% foetal bovine serum (HyClone, GE Healthcare Bio-Sciences, Pittsburgh, PA, USA) at 37°C and 5% CO_2_ in humidified air. The myoblasts were maintained in growth medium until 80% confluence was reached (day 0). Thereafter, growth medium was changed to DMEM with 2% horse serum (Thermo Fisher Scientific, Waltham, MA, USA) for differentiation. The differentiation medium was renewed daily for 5 days (day 5). When multinucleated myotubes were obvious by microscopy, we treated them with L-sodium LA (Sigma-Aldrich, Darmstadt, Germany) at 8 mM or vehicle alone for 1 or 24 h. This concentration of LA is observed in human blood after high-intensity exercise and has been previously reported in the literature [2, 20, 21, 22, 23].

### 2.2 Western blotting

Western blot analyses were carried out as described previously [24]. Immediately after each treatment, cells were washed twice with ice-cold phosphate-buffered saline, and then lysed in ice-cold radioimmunoprecipitation buffer (50 mM Tris-HCl, 1% NP40, 150 mM NaCl, 1 mM ethylenediaminetetraacetic acid and 0.1% sodium dodecyl sulphate (SDS)) supplemented with protease inhibitors and phosphatase inhibitors (Sigma-Aldrich) to prepare whole cell lysates. These were sonicated to homogenize the cell suspension and centrifuged at 13,000 *g* for 10 min to remove cell debris. Protein concentrations in the supernatant were measured using a BCA protein assay kit (Pierce, Rockford, IL, USA); aliquots of denatured protein (30 μg) were loaded on SDS-polyacrylamide (11%) gels, and transferred to a nitrocellulose membrane (Pall Corporation, New York, NY, USA) in transfer buffer (25 mM Tris-HCl, 192 mM glycine and 20% methanol). The membrane was blocked with 5% milk in Trisbuffered saline containing 0.2% Tween 20 (TBS-T) for 1.5 h at room temperature (RT) and incubated with 5% bovine serum albumin in TBS-T with specific antibodies overnight at 4°C (Table 1). After washing three times in TBS-T, the membranes were incubated with horseradish peroxidase-conjugated secondary antibodies for 2 h at RT. Specific bands were detected using an enhanced chemiluminescence detection system (Pierce) and band intensities were quantified using a Chemidoc Touch Image system and Quantity One® 4.6 image analysis software (Bio-Rad Laboratories, Inc., Hercules, CA, USA).

**Table 1:** Antibodies used in this study.

Antibodies	Source	Manufacturer	Catalog#	Dilution
anti-p**-**ACC Ser79	Rabbit	Cell Signaling	#3661	1:1000
anti-ACC	Rabbit	Cell Signaling	#3662	1:1000
anti-p-AMPK Thr172	Rabbit	Cell Signaling	#2535	1:1000
anti-AMPK	Rabbit	Cell Signaling	#2532	1:1000
anti-p-mTOR Ser2448	Rabbit	Cell Signaling	#2971	1:1000
anti-mTOR	Rabbit	Cell Signaling	#2972	1:1000
anti-p-ratpor Ser792	Rabbit	Cell Signaling	#2083	1:1000
anti-raptor	Rabbit	Cell Signaling	#2280	1:1000
anti-p-Akt Ser473	Rabbit	Cell Signaling	#9271	1:1000
anti-Akt	Rabbit	Cell Signaling	#9272	1:1000
anti-p-FOXO1 Thr24/FoxO3a Thr32	Rabbit	Cell Signaling	#9464	1:1000
anti-p-P70S6K Thr389	Mouse	Cell Signaling	#9206	1:1000
anti-P70S6K	Rabbit	Cell Signaling	#9202	1:1000
anti-atrogin1	Mouse	Santa Cruz Biotechnology	sc-166806	1:500
anti-myo-D	Rabbit	Santa Cruz Biotechnology	sc-304	1:500
anti-myogenin	Mouse	Santa Cruz Biotechnology	sc-12732	1:500
anti-SirT1	Rabbit	Cell Signaling	#9475	1:1000
anti-PGC1α	Rabbit	Abcam	ab54481	1:1000
Anti-Lamin B	Mouse	Santa Cruz	Sc-7597	1:1000
anti-α-actinin	Mouse	Santa Cruz Biotechnology	sc-166524	1:1000
anti-β-actin	Mouse	Prosci	PM-7547	1:10000

### 2.3 Measurement of myotube diameters

To measure the effects of LA on myotube diameters, images of myotubes after treatments with LA for the indicated times were visualized at 20 × magnification using phase contrast light microscopy (Leica Microsystems, Wetzlar, Germany). The diameters of all myotubes (n = 21 – 30) in randomly selected microscope fields from six wells for each treatment condition were measured using the National Institutes of Health software ImageJ v. 1.38x (NIH, Bethesda, MD, USA; https://imagej.nih.gov/ij/),and the means were compared. Following the instruction, we determined the myotube diameter by measuring the length of a line perpendicular to the edge of selected myotubes.

### 2.4 Preparation of subcellular fraction

Total cell lysates were obtained by scraping the cells with nuclear extraction buffer 1 (NEB1) containing 10 mM HEPES, 10 mM KCl, 0.1 mM EDTA, 0.1 mM EGTA, protease inhibitors and phosphatase inhibitors, followed by incubation on ice for 10 min. After centrifugation at 16,000 x g for 30 seconds, supernatant was collected as non-nuclear fraction. The pellet was washed with NEB1 followed by the incubation with NEB2 (20 mM HEPES, 0.4 M NaCl, 1 mM EDTA, 1 mM EGTA, protease inhibitors and phosphatase inhibitors) on ice for 15 min. After centrifugation, the supernatant was collected as nuclear fraction.

### 2.5 Statistics

Data are presented as the mean ± standard deviation (SD) of western blot band intensities (n = 3) or myotubue diameters. The effects of LA on the expression levels of proteins or the diameters of C2C12 myotubes were compared using unpaired two-tail *t* tests using GraphPad Prism (version 6.0; GraphPad Software, San Diego, CA, USA). We assumed *P* < 0.05 to be significant.

## 3 Results

### 3.1 Early responses of the AMPK signaling pathway to LA overload

**Figure 1:**
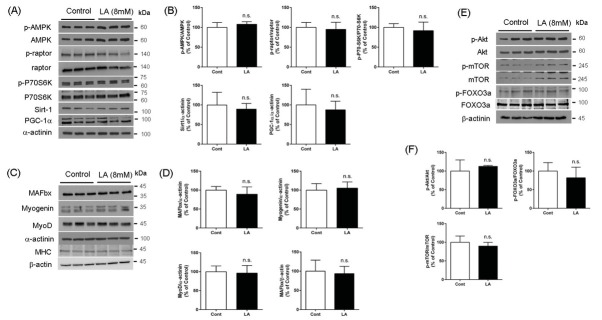
Early response of factors associated with myogenesis or atrophy to LA overload. The levels of protein expression in myotubes treated with lactate (LA, 8 mM) for 1 h were similar to the control myotubes (A, C, E). The quantified band intensities showed no significant difference (B, D, F). The mean band intensities quantified from two independent experiments (n=3) were compared, and representative bands are presented.

AMPK, a master regulator of cellular energy metabolism, is crucial for myogenesis and protein synthesis [25]. To evaluate the effect of LA on AMPK and its downstream signaling proteins, we evaluated the levels of phosphorylated AMPK at Thr172 (p-AMPK), peroxisome proliferator-activated receptor gamma coactivator-1 alpha (PGC-1α), Sirtuin 1 (Sirt1), phosphorylated raptor at Ser792 (p-raptor) and phosphorylated P70-S6K at Thr421/Ser424 (p-P70-S6K). As shown in Figures 1A and 1B, treatment of C2C12 myotubes with LA at 8 mM for 1 h, no significant difference in the levels of p-AMPK or its downstream signaling proteins was seen when compared with vehicle controls.

### 3.2 Early responses of myogenenic proteins and Akt-FOXO3a pathway to LA overload

Activation of the PI3K/Akt signaling pathway represents another canonical pathway of skeletal muscle hypertrophy, and is a key regulator of protein synthesis and myogenesis via the mTORC1–P70-S6K pathway and FOXO3a, respectively[26, 27]. Akt inhibits translocation of cytosolic FOXO3a to the nucleus by phosphorylation, which inhibits the expression of an atrophic transcription factor, MAFbx [27, 28]. When we treated myotubes with LA for 1 h, the activity of Akt (estimated with the level of phosphorylated Akt at Ser473), phosphorylated mammalian target of rapamycin (mTOR) Ser2448 (p-mTOR) and phosphorylated FOXO3a at Thr24/32 (p-FOXO3a) were similar to the levels in control myotubes (Fig. 1E, F). To evaluate the short-term effect of LA on the muscle differentiation signals involved in myogenesis and muscle atrophy, we tested the levels of MyoD, myogenin and myosin heavy chain (MHC) for myogenic signals and MAFbx (also known as atrogin-1) for atrophic signals, respectively [29, 30]. When we treated C2C12 myotubes with LA at 8 mM for 1 h to evaluate the early response, the levels of MyoD, myogenin and MHC were similar to those in cells treated with vehicle alone. In addition, the level of MAFbx—an atrophic transcription factor—in LA-treated myotubes was not significantly different from controls (Fig. 1C, D).

### 3.3 Delayed activation of AMPK signalling pathways by LA overload

**Figure 2:**
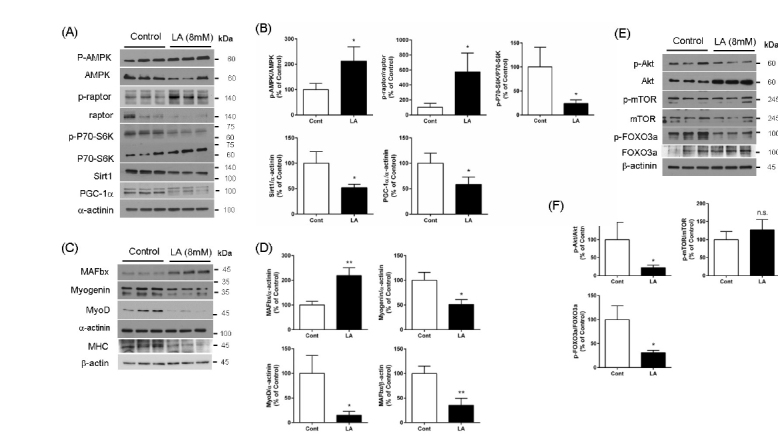
Delayed response of myogenic or atrophic factors to LA overload. The levels of AMPK-mediated inhibition of mTOR (decreased p-raptor and p-P70-S6K) and of Sirt1 and PGC-1α in myotubes treated with lactate (LA, 8 mM) for 24 h were significantly different when compared to control myotubes (A, B). Myogenic factors (myogenin, MyoD and MHC) in LA-treated myotubes were significantly lower, while atrophic factor (MAFbx) was significantly higher than control myotubes (C, D). Akt-mediated inhibition (phosphorylation) of FOXO3a was significantly blocked by LA treatment for 24 h (E, F). The mean band intensities quantified from two independent experiments (n=3) were compared, and representative bands were presented. *p<0.05, **p<0.01 by unpaired t-test.

As shown in Figures 2A and 2B treatment of C2C12 myotubes with LA (8 mM) for 24 h significantly increased the level of p-AMPK but significantly suppressed the expressions of Sirt1 and PGC-1α. The level of p-raptor, a downstream target of AMPK in the mTOR complex, was increased significantly, while the level of p-P70-S6K was suppressed significantly by LA treatment for 24 h.

### 3.4 Inhibition of Akt-mediated myogenic signaling pathways by LA overload for 24 h

**Figure 3:**
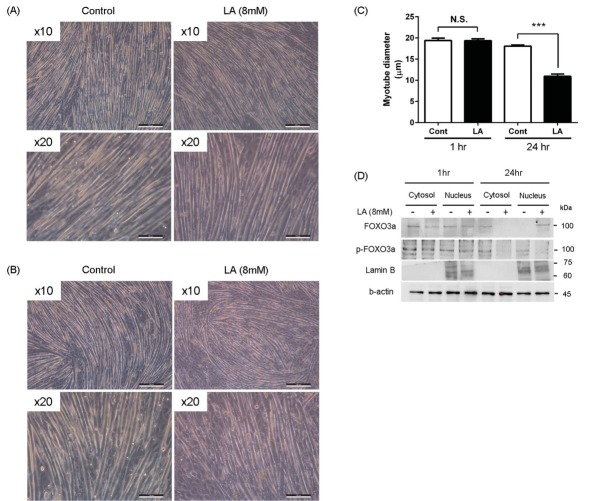
Reduction of myotube diameters by LA. (A – C) When we treated myotubes with lactate (LA, 8 mM) for 24 h, mean diameter of LA treated myotubes was significantly lower than control myotubes. ***p<0.001 vs. control (Cont) by unpaired t-test. (D) When we prepared the subcellular fraction (for details, see Method 2.4.), LA treatment for 24 h increased the nuclear translocation of FOXO3a, while LA overload for 1 h did not change the level of nuclear FOXO3a.

Treatment of C2C12 myotubes with LA for 24 h significantly inhibited the levels of p-Akt and downstream targets of p-FOXO3a, while the expression of p-mTOR was similar to the controls. Consistent with the suppression of Akt-mediated myogenic pathways, we observed that LA overload significantly suppressed the levels of MyoD, myogenin and MHC, but increased the level of MAFbx, when compared with vehicle-treated controls. Consistent with the results of anti-myogenic effects of LA, we also observed that LA treatment for 24 h decreased the mean myotube diameter (Fig. 3B, 3C). Furthermore, we observed the treatment of LA for 24 h, but not for 1hr, increased the nuclear translocation of FOXO3a, a transcription factor for MAFbx (Fig 3D), indicating that LA-induced nuclear translocation of FOXO3a may be a mechanism of the decreased diameter of myotube by LA.

## 4 Discussion

In pathological conditions, including but not limited to cellular hypoxia [31] and cancer [5] that induce increased LA production, or hepatic failure [32], diabetes [33] and biguanide intoxication [34] that inhibit hepatic LA clearance, the levels of LA can be elevated in blood and skeletal muscle. LA can be used as an energy source instead of pyruvate; however, LA can also act as a signaling molecule [10, 35, 36]. Here we tested the effects of LA overload on myogenic signaling using differentiated C2C12 myotubes. We found that LA overload for 24 h—but not for 1 h—significantly suppressed the levels of Akt-mediated myogenic signaling proteins p-P70-S6K, MyoD, myogenin, Sirt1 and PGC-1α, while it increased the levels of the anti-myogenic molecules p-AMPK, FOXO3a and MAFbx.

In skeletal muscle, Akt activates the mTOR/P70-S6K protein synthesis pathway that induces muscle hypertrophy [26, 27]. In addition, Akt inhibits the transcriptional activity of FOXO3a by phosphorylation [26] followed by inhibition of MAFbx expression. AMPK can also phosphorylate FOXO3a, but unlike Akt, AMPK-mediated phosphorylation increases the activity of this target molecule [37, 38]. Here, we observed that LA overload for 24 h significantly increased the activity of AMPK but suppressed Akt activity, indicating that LA accumulation for longer times can initiate the canonical anti-myogenic signaling pathways. Consistent with the effects of LA on AMPK and Akt, we observed that LA induced the expression of MAFbx, a target molecule of FOXO3a, but decreased the myogenic transcription factors, MyoD and myogenin. Phenotypically, myotubes treated with LA for 24 h, but not in cells treated for 1 h, showed the significantly lower mean myotube diameter than control myotubes. The decreased myotube diameter by treatment of LA for 24 h was consistent with the decreased expression of MHC (Fig. 2C and D) and increased nuclear translocation of FOXO3a (Fig. 3D), which further supports the muscle atrophic effects of chronic LA overload. Protein synthesis is essential for increasing skeletal muscle mass [39]. AMPK activation inhibits mTORC1-mediated protein synthesis in which P70-S6K activity is crucial [13, 40, 41]. We observed that LA treatment inhibited the phosphorylation of P70-S6K, consistent with increased levels of p-AMPK and p-raptor, therefore, LA overload might inhibit the protein synthesis pathway required for hypertrophy, although we did not study the protein synthesis rate specifically. We could not detect the change of mTOR phosphorylation by LA treatment, however, LA-induced AMPK activation may inhibit mTORC1-mediated protein synthetic activity through the phosphorylation of raptor at Ser792 rather than direct mTOR phosphorylation at Ser2448. Sirt1, a deacetylase, inhibits the myogenic molecules MyoD and MEF2 by formation of inhibitory complexes [42], even though this has not been properly tested in vivo. However, in contrast, a recent study provided evidence that Sirt1 and PGC-1α can induce the levels of myogenic factors in conjunction with MyoD [17, 43]. Although further studies in the detailed mechanisms and roles of Sirt1 and PGC-1α in LA overload are required, our data suggest that LA overload for long durations can inhibit the myogenic roles of Sirt1 and PGC-1α, in addition to Akt-mediated myogenic signaling. It is unlikely that the LA-mediated activation of AMPK is linked to increased activity of Sirt1 and PGC-1α, which are essential in mitochondrial biogenesis [25]. Skeletal muscle contraction by exercise activates AMPK by increasing AMP/ATP ratios [41]. However, in our experimental conditions we treated myotubes with a high concentration of LA without glucose deprivation, so this did not mimic exercise but rather the accumulation of LA. Otherwise, during exercise, LA build-up might activate AMPK independent of the AMP/ATP ratio. In addition, because LA overload suppressed the expression of both Sirt1 and PGC-1α in this study, the effects of LA on mitochondrial biogenesis should be evaluated further.

In skeletal muscle, Akt activates the mTOR/P70-S6K protein synthesis pathway that induces muscle hypertrophy [26, 27]. In addition, Akt inhibits the transcriptional activity of FOXO3a by phosphorylation [26] followed by inhibition of MAFbx expression. AMPK can also phosphorylate FOXO3a, but unlike Akt, AMPK-mediated phosphorylation increases the activity of this target molecule [37, 38]. Here, we observed that LA overload for 24 h significantly increased the activity of AMPK but suppressed Akt activity, indicating that LA accumulation for longer times can initiate the canonical anti-myogenic signaling pathways. Consistent with the effects of LA on AMPK and Akt, we observed that LA induced the expression of MAFbx, a target molecule of FOXO3a, but decreased the myogenic transcription factors, MyoD and myogenin. Phenotypically, myotubes treated with LA for 24 h, but not in cells treated for 1 h, showed the significantly lower mean myotube diameter than control myotubes. The decreased myotube diameter by treatment of LA for 24 h was consistent with the decreased expression of MHC (2C, and D), and increased nuclear translocation of FOXO3a (Fig. 3D), which further supports the muscle atrophic effects of chronic LA overload. Protein synthesis is essential for increasing skeletal muscle mass [39]. AMPK activation inhibits mTORC1-mediated protein synthesis in which P70-S6K activity is crucial [13, 40, 41]. We observed that LA treatment inhibited the phosphorylation of P70-S6K, consistent with increased levels of p-AMPK and p-raptor, therefore, LA overload might inhibit the protein synthesis pathway required for hypertrophy, although we did not study the protein synthesis rate specifically. We could not detect the change of mTOR phosphorylation by LA treatment, however, LA-induced AMPK activation may inhibit mTORC1-mediated protein synthetic activity through the phosphorylation of raptor at Ser792 rather than direct mTOR phosphorylation at Ser2448. Sirt1, a deacetylase, inhibits the myogenic molecules MyoD and MEF2 by formation of inhibitory complexes [42], even though this has not been properly tested in vivo. However, in contrast, a recent study provided evidence that Sirt1 and PGC-1α can induce the levels of myogenic factors in conjunction with MyoD [17, 43]. Although further studies in the detailed mechanisms and roles of Sirt1 and PGC-1α in LA overload are required, our data suggest that LA overload for long durations can inhibit the myogenic roles of Sirt1 and PGC-1α, in addition to Akt-mediated myogenic signaling. It is unlikely that the LA-mediated activation of AMPK is linked to increased activity of Sirt1 and PGC-1α, which are essential in mitochondrial biogenesis [25]. Skeletal muscle contraction by exercise activates AMPK by increasing AMP/ATP ratios [41]. However, in our experimental conditions we treated myotubes with a high concentration of LA without glucose deprivation, so this did not mimic exercise but rather the accumulation of LA. Otherwise, during exercise, LA build-up might activate AMPK independent of the AMP/ATP ratio. In addition, because LA overload suppressed the expression of both Sirt1 and PGC-1α in this study, the effects of LA on mitochondrial biogenesis should be evaluated further.

**Figure 4:**
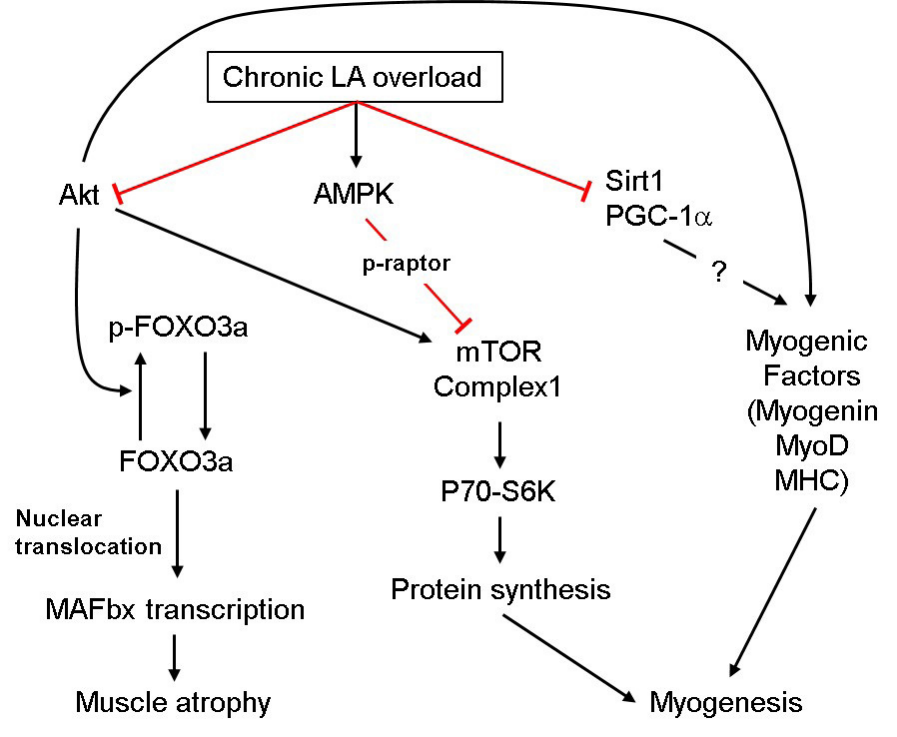
Summarized effects of chronic LA overload on the signal proteins regulating myogenesis and muscle atrophy. Red-colored lines and black lines indicate the inhibitory and activating effects, respectively.

There are several limitations of our study; first, the use of a murine cell-line rather than use of human primary cells to evaluate the effects of LA is a limitation. C2C12 differentiated myotube model is a widely used experimental tool for the experiment of muscle biology. Nevertheless, our results should be confirmed in human primary cell culture model. Second, we did not perform experiments for the effects of LA at intermediate time (e.g., 4 h, 12 h). However, the aim of our study has focused on the acute effects of LA during short duration that mimic the strenuous exercise and chronic effects of LA that mimic a pathologic LA accumulating condition. Finally, we could not confirm the effects of LA-induced activation of AMPK on myogenic signaling, but we speculate LA-mediated inhibition of myogenesis through AMPK-mTORC1-P70-S6K pathways. Fu et al. reported contradictory result that knockdown of AMPK-α1 inhibited myogenic signaling [15, 17], the effects of LA-mediated AMPK activation on the myogenenic signaling should be further investigated, although we recently reported that inflammation activated AMPK and inhibition of the expression of myogenic signaling [44].

In conclusion, LA overload for 24 h inhibited myogenic pathways through the activation of AMPK but also via suppression of Akt, which is consistent with decreased levels of myogenic factors (MyoD and myogenin) and increased levels of the atrophic factor MAFbx. The LA-mediated anti-myogenesis, at least in part, is likely to induce transcriptional activity of FOXO3a. Our study was performed under in vitro experimental conditions using C2C12 myotubes, so further in vivo evidence is needed to support our results.
